# Analysis of cardiac motion without respiratory motion for cardiac stereotactic body radiation therapy

**DOI:** 10.1002/acm2.13002

**Published:** 2020-09-12

**Authors:** Zi Ouyang, Paul Schoenhagen, Oussama Wazni, Patrick Tchou, Walid I. Saliba, John H. Suh, Ping Xia

**Affiliations:** ^1^ Department of Radiation Oncology Taussig Cancer Institute Cleveland Clinic Cleveland OH USA; ^2^ Department of Radiology Imaging Institute Cleveland Clinic Cleveland OH USA; ^3^ Department of Cardiovascular Medicine Miller Family Heart & Vascular Institute Cleveland Clinic Cleveland OH USA

**Keywords:** 4D‐CT, cardiac substructures, SBRT, ventricular tachycardia

## Abstract

**Purpose/objective(s):**

To study the heart motion using cardiac gated computed tomographies (CGCT) to provide guidance on treatment planning margins during cardiac stereotactic body radiation therapy (SBRT).

**Materials/methods:**

Ten patients were selected for this study, who received CGCT scans that were acquired with intravenous contrast under a voluntary breath‐hold using a dual source CT scanner. For each patient, CGCT images were reconstructed in multiple phases (10%–90%) of the cardiac cycle and the left ventricle (LV), right ventricle (RV), ascending aorta (AAo), ostia of the right coronary artery (O‐RCA), left coronary artery (O‐LCA), and left anterior descending artery (LAD) were contoured at each phase. For these contours, the centroid displacements from their corresponding average positions were measured at each phase in the superior–inferior (SI), medial–lateral (ML), and anterior–posterior (AP). The average volumes as well as the maximum to minimum ratios were analyzed for the LV and RV.

**Results:**

For the six contoured substructures, more than 90% of the measured displacements were <5 mm. For these patients, the average volumes ranged from 191.25 to 429.51 cc for LV and from 91.76 to 286.88 cc for RV. For each patient, the ratios of maximum to minimum volumes within a cardiac cycle ranged from 1.15 to 1.54 for LV and from 1.34 to 1.84 for RV.

**Conclusion:**

Based on this study, cardiac motion is variable depending on the specific substructure of the heart but is mostly within 5 mm. Depending on the location (central or peripheral) of the treatment target and treatment purposes, the treatment planning margins for targets and risk volumes should be adjusted accordingly. In the future, we will further assess heart motion and its dosimetric impact.

## 1 INTRODUCTION

Ventricular tachycardia (VT) is a cardiac arrhythmia defined as 3 or more consecutive complexes in duration emanating from the ventricles at a rate > 100 bpm (cycle length: <600 ms).^1^ It may occur in individuals of all age groups, often due to a structural cardiac abnormality or a recognized predisposing condition. However, VT may also be found idiopathic in some patients.^2^ A dangerous condition related to VT is ventricular fibrillation. With ventricular fibrillation, the diseased ventricle contracts in a very rapid and uncoordinated manner, resulting in heart failure, frequent fainting episodes, or sudden death by a cardiac arrest. Sustained ventricular arrhythmia is the most common cause of sudden cardiac death, accounting for 75–80% of cases.^3^ Implantable cardioverter‐defibrillators (ICDs) have been used, along with conventional and antiarrhythmic drug therapy, to prevent sudden cardiac arrest in patients with depressed left ventricular function. Ventricular tachycardia has a high recurrence rate, and catheter ablation can be used to prevent or reduce recurrent episodes of VT.^4^, ^5^ However, its invasive nature increases the risk of procedural complications.^6^

A recent publication from Washington University in St. Louis reported a noninvasive cardiac radiation for ablation of VT with promising initial results — in five patient with refractory VT, they reported a markedly reduction of VT.^7^ Although this innovative treatment is not a current standard of care, it can be offered to patients who have treatment‐refractory VT and have limited other treatment options. The radioablation procedure, referred to as stereotactic radiosurgery, has been a mainstay of treatment for non‐malignant conditions such as trigeminal neuralgia and arteriovenous malformations. In the thorax, the radioablation procedure, referred as stereotactic body radiation therapy (SBRT), has been applied for patients with early‐stage lung cancers, who are medically inoperable.^8^ As reported by Cuculich et al.,^7^ SBRT was used as the noninvasive treatment method instead of catheter radiofrequency ablation to treat recurrent VT.

In SBRT, organ motion management is important. In the thorax and abdominal regions, breathing motion managements are well studied.^9^, ^10^ Breath‐hold methods have been applied to patients with lung cancer, liver tumors, or breast cancer during SBRT and conventional radiotherapy. Comparing to the breathing motion, cardiac motion is secondary. If breathing motion can be minimized using either breath‐hold method or breathing gated treatment method, the planning margin for cardiac radiation ablation can be drastically reduced. Studies of cardiac motion in radiation therapy are scarce. Different from the breathing motion, cardiac motion is fast and asymmetrical.^11^, ^12^, ^13^ The motion magnitude of each substructure of the heart varies in the rapid cycle of heart beat. Typical CT scanners used in radiation oncology departments are not suitable for study of the cardiac motion. In this work, separated from the impact of the breathing motion, we studied the heart substructure motion using gated cardiac computed tomography (CT) images acquired in the cardiovascular department of our institution to seek for planning margin guidance for cardiac SBRT treatment.

Recent studies showed that the doses to the substructures of heart, especially the left ventricle and the left anterior descending artery, were predictive to radiotherapy related cardiac toxicity.^14^, ^15^, ^16^, ^17^ The traditional treatment planning constraint, mean dose to whole heart, is not as good a predictor and does not correlate with the doses to the substructures. Therefore, studying the motion of the heart substructures is essential to the safety of cardiac SBRT treatment. This study analyzes the motion of the following substructures: the left ventricle (LV), right ventricle (RV), ascending aorta (AAo), ostium of the left coronary artery (O‐LCA), ostium of the right coronary artery (O‐RCA), and the left anterior descending artery (LAD).

## 2 MATERIALS AND METHODS

This retrospective study is approved by our local Institutional Review Board. Ten patients who received cardiac gated CT (CGCT) scans in the context of transcatheter aortic valve replacement (TAVR) were selected for this study. The CGCT Images were acquired while patients were under a voluntary breath‐hold using a dual source CT scanner (Siemens Definition Force dual‐source scanner, Malvern, PA). Intravenous contrast was administered (60 mL iodinated contrast with flow rate of 4 mL/s), and retrospectively gated reconstructions were performed. We used bolus tracking in the descending aorta (threshold of 100 HU). The reference tube current and tube voltage were set to 288 mAs and 100 kV, respectively. Cardiac gated CT images were reconstructed with 1 mm slice thickness in multiple phases (10%–90%) of the cardiac cycle. The average Dose Length Product (DLP) was 617 ± 232 mGy × cm.

**Fig. 1:**
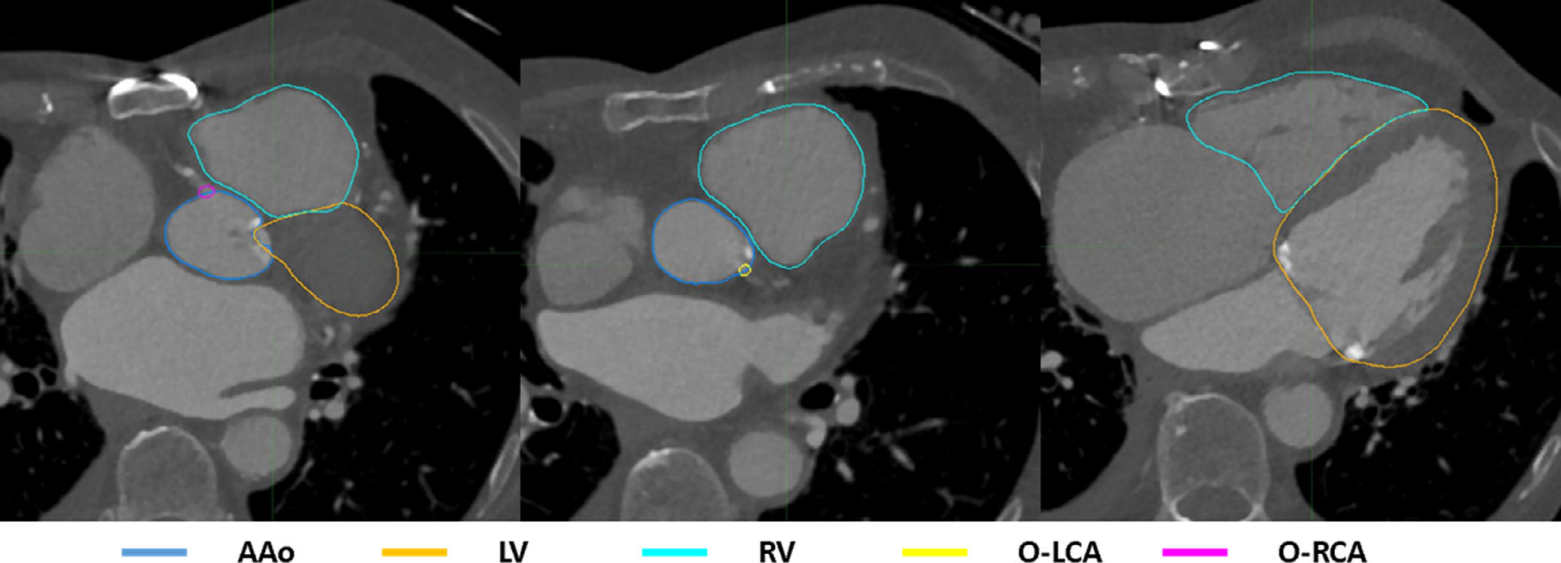
Example contours of a patient in 10% phase. Blue: AAo; Orange: LV; Cyan: RV; Yellow: O‐LCA; Magenta: O‐RCA. (AAo: ascending aorta; LV: left ventricle; RV: right ventricle; O‐LCA: ostium of left coronary artery; O‐RCA: ostium of right coronary artery).

**Fig. 2:**
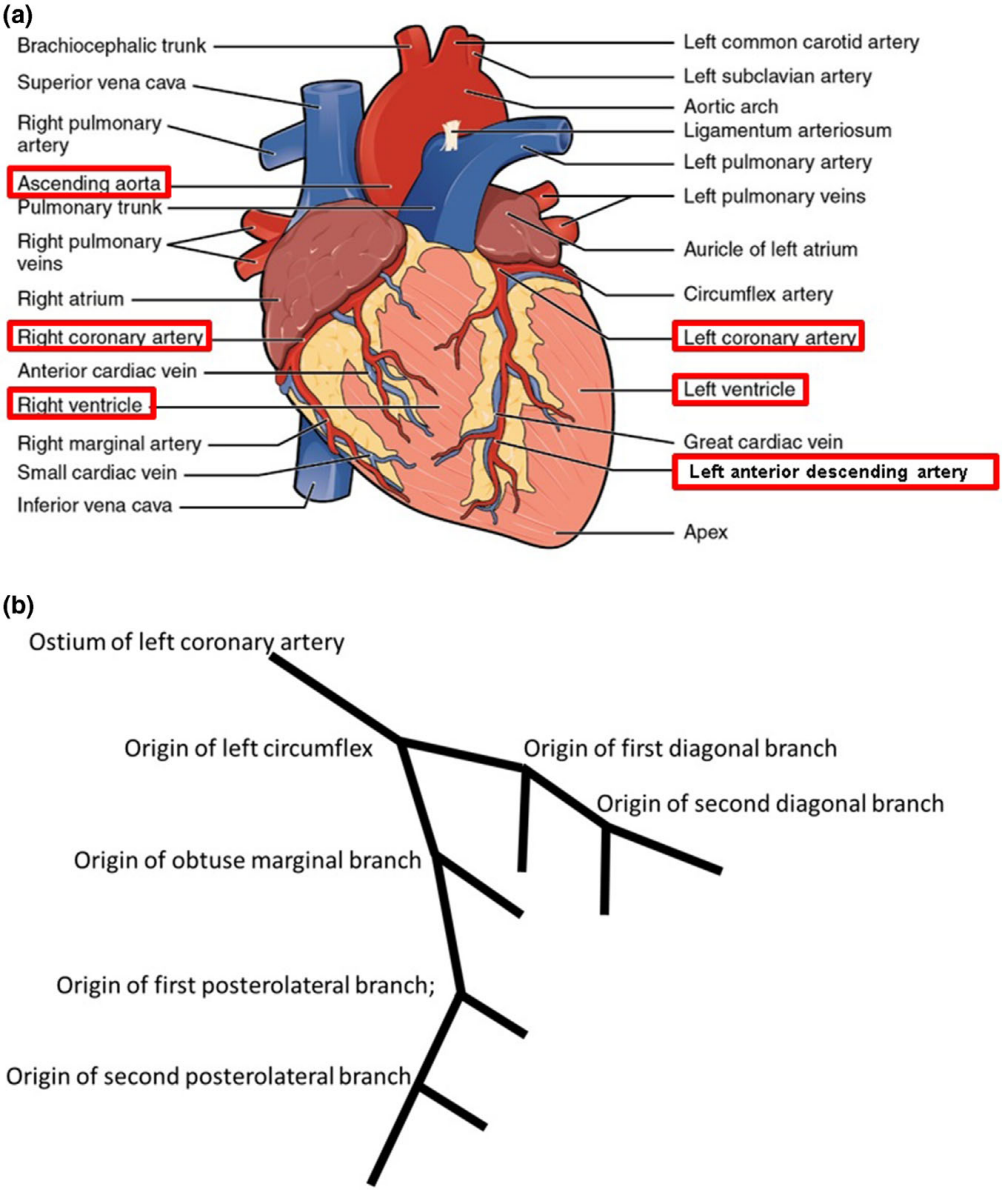
(a) A picture of an external anatomy of the heart inside the pericardium. Adapted from Heart Anatomy in Anatomy and Physiology, by Betts et al., retrieved from https://openstax.org/books/anatomy-and-physiology/pages/19-1-heart-anatomy Copyright 2020 by OpenStax 20. (b) A schematic of the bifurcations points of the left coronary artery.

For each patient, the left ventricle (LV), right ventricle (RV), ascending aorta (AAo), ostia of the right coronary artery (O‐RCA), left coronary artery (O‐LCA), and left anterior descending coronary artery (LAD) were contoured at each phase.^18^ Figure 1 shows the example contours of these structures. To study the motion of the left anterior descending coronary artery (LAD), instead of contouring the entire LAD, we used an alternative method based on a published study.^19^ The coronary arteries can be described by landmarks such as openings and bifurcation points. As shown in Figs. 2(a)^20^ and 2(b), the left main bifurcates into the left anterior descending and the left circumflex. The ostium of the left coronary artery and the origin of the first diagonal branch were contoured. The midpoint of these two structures were calculated, and its motion was used to represent the motion of the LAD.

The CGCT images were contoured and analyzed in MIM (MIM Software Inc., Cleveland, OH). The positional coordinates of the centroid of each contour were recorded in the superior–inferior (SI), medial–lateral (ML), and anterior–posterior (AP) directions. For each contour, an average position was calculated by averaging the positional values in all nine phases. The absolute difference from the position in each phase to the average position was labeled as absolute displacement. For example, *SI_n_* (*n* = 1, 2, …, 9) was the SI coordinate at the *nth* phase, and *SI_0_* was the average of *SI_n_*. The absolute displacement was therefore |*SI_n_* − *SI_0_*| for the *nth* phase.

The distance to average position for each phase is defined as shown in Eq. (1), where *d* is the distance; *SI*, *ML*, and *AP* are the position values; *n* is the phase number.

(1) $$d=\sqrt{{\left(SI_{n}‐SI_{0}\right)}^{2}+{\left(ML_{n}‐ML_{0}\right)}^{2}+{\left(AP_{n}‐AP_{0}\right)}^{2}}$$

For data analysis, this work reports the mean value ± standard deviation (SD), minimum, maximum, as well as the distributions of the absolute displacement and distance with 0.5 or 1 mm intervals.

To study the volume changes of the LV and RV, the contour volumes were recorded for each patient in each phase. The average volumes were calculated for the LV and RV for each patient by averaging over the nine phases. The ratios of maximum to minimum volumes were also calculated for each patient.

## 3 RESULTS

For the 10 patients, as shown in Table 1, the average absolute displacements for the LV were 0.51 ± 0.53 mm in the SI direction, 0.53 ± 0.43 mm in the ML direction, 1.20 ± 0.83 mm in the AP direction; for RV, 1.00 ± 0.79 mm in the SI direction, 1.87 ± 1.47 mm in the ML direction, 0.54 ± 0.45 mm in the AP direction; for AAo, 0.48 ± 0.38 mm in the SI direction, 0.96 ± 0.78 mm in the ML direction, 1.30 ± 0.84 mm in the AP direction; for O‐RCA, 0.97 ± 0.83 mm in the SI direction, 1.64 ± 1.17 mm in the ML direction, 1.52 ± 1.00 mm in the AP direction; for O‐LCA, 1.06 ± 0.82 mm in the SI direction, 1.24 ± 0.87 mm in the ML direction, 1.21 ± 0.85 mm in the AP direction; for LAD, 1.17 ± 0.91 mm in the SI direction, 1.22 ± 0.86 mm in the ML direction, and 1.08 ± 0.71 mm in the AP direction. The maximum displacement was 7.13 mm, which was observed for the RV. The average displacements were within 2 mm for all contoured structures.

**Table 1:** Absolute displacements of the centroids of the left ventricle (LV), right ventricle (RV), ascending aorta, ostia of the left and right coronary arteries, and the derived position of the left anterior descending artery in a cardiac cycle for ten patients.

	Mean (mm)	SD (mm)	Min (mm)	Max (mm)
LV				
SI	0.51	0.53	0.00	3.24
ML	0.53	0.43	0.00	2.32
AP	1.20	0.83	0.04	3.44
RV				
SI	1.00	0.79	0.01	3.70
ML	1.87	1.47	0.02	7.13
AP	0.54	0.45	0.00	2.34
AAo				
SI	0.48	0.38	0.01	1.95
ML	0.96	0.78	0.02	3.43
AP	1.30	0.84	0.09	3.14
O‐RCA				
SI	0.97	0.83	0.00	3.72
ML	1.64	1.17	0.11	4.96
AP	1.52	1.00	0.02	3.92
O‐LCA				
SI	1.06	0.82	0.02	3.32
ML	1.24	0.87	0.02	3.66
AP	1.21	0.85	0.01	3.16
LAD				
SI	1.17	0.91	0.05	3.63
ML	1.22	0.86	0.03	3.65
AP	1.08	0.71	0.01	2.85

**Fig. 3:**
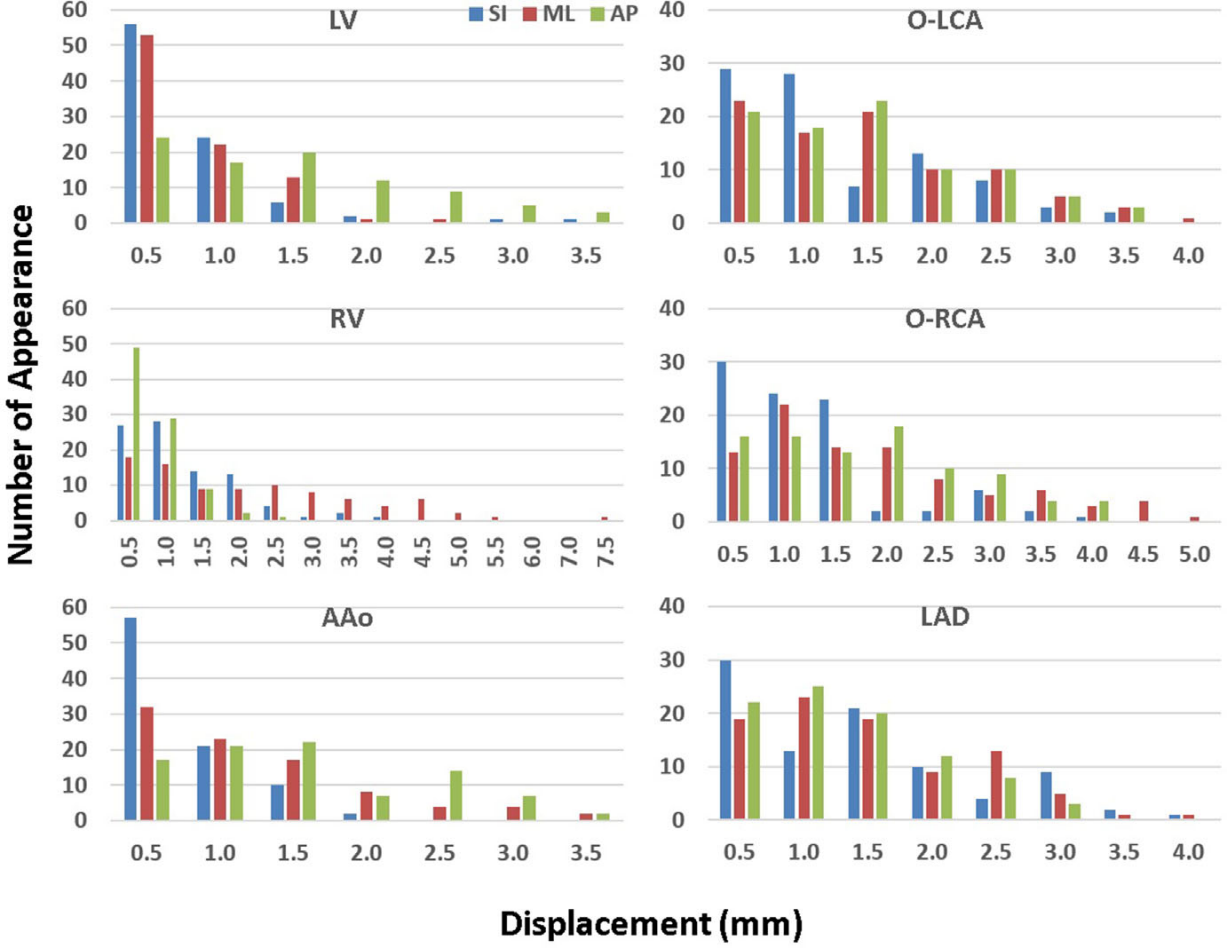
Number of displacements within each half mm range in the superior–inferior, medial–lateral, and anterior–posterior directions for the centroids of the LV, RV, AAo, O‐RCA, O‐LCA, and LAD. (AAo: ascending aorta; LV: left ventricle; RV: right ventricle; O‐LCA: ostium of left coronary artery; O‐RCA: ostium of right coronary artery; LAD: left anterior descending artery).

Figure 3 shows the histograms of the absolute displacements of the centroid of each structure within the defined motion ranges. For the LV, motion in the AP direction was larger than that in the SI and ML direction. For the RV, the largest motion was observed to be in the ML direction. For the AAo, the SI direction was the most stable direction. Motions for the O‐LCA, O‐RCA, and LAD did not show directional preference.

**Fig. 4:**
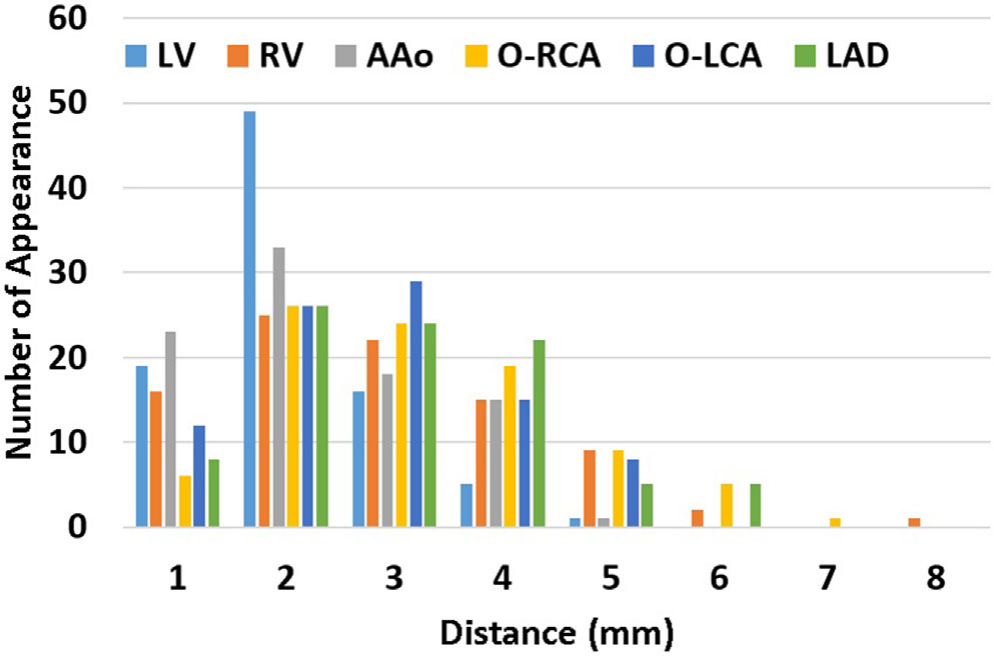
Number of distances within each range for the centroids of the LV, RV, AAo, O‐RCA, O‐LCA, and LAD. (AAo: ascending aorta; LV: left ventricle; RV: right ventricle; O‐LCA: ostium of left coronary artery; O‐RCA: ostium of right coronary artery; LAD: left anterior descending artery).

Table 2 shows the average distance for the centroid of each structure as calculated using Eq. (1), and Fig. 4 shows the histograms of the distances within the defined motion ranges. While the majority of the distances were <5 mm, the RV, O‐RCA, and LAD had slightly larger motions.

**Table 2:** Distance of ROI centroid position in each phase to its average position. (AAo: ascending aorta; LV: left ventricle; RV: right ventricle; O‐LCA: ostium of left coronary artery; O‐RCA: ostium of right coronary artery; LAD: left anterior descending artery).

	Mean (mm)	SD (mm)	Min (mm)	Max (mm)
LV	1.56	0.83	0.31	4.54
RV	2.40	1.40	0.43	7.85
AAo	1.83	0.97	0.20	4.28
O‐RCA	2.69	1.30	0.35	6.51
O‐LCA	2.26	1.08	0.26	4.73
LAD	2.49	1.21	0.29	5.45

**Fig. 5:**
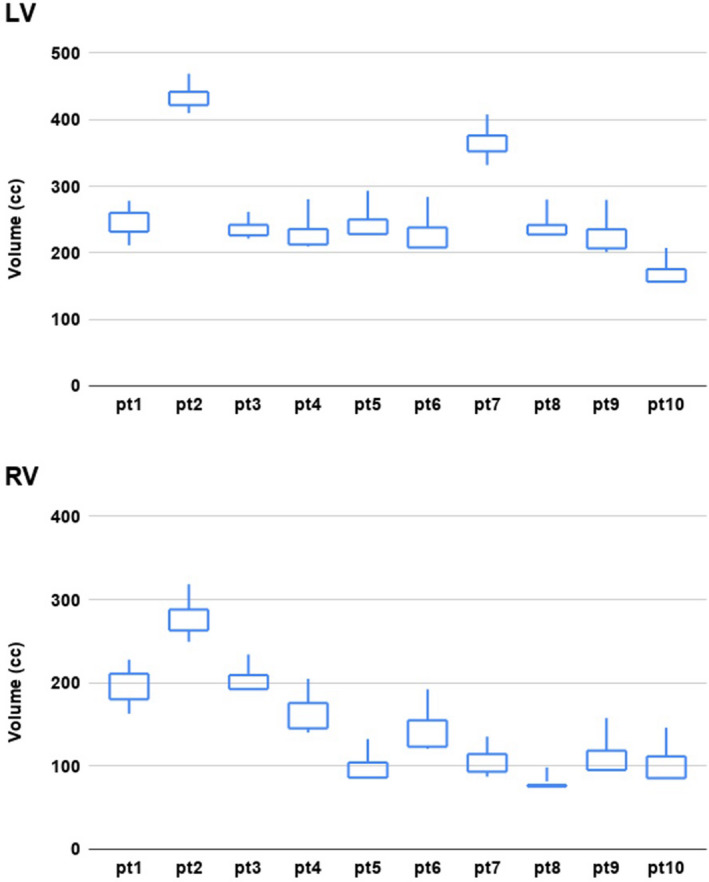
Candlestick charts of the LV and RV volumes for each patient in the imaged cardiac phases. Volumes of the minimum, maximum, 10% phase, and 90% phase are shown in each candlestick. (LV: left ventricle; RV: right ventricle).

As shown in Table 3, the ratio of the maximum phase volume to the minimum phase volume ranged from 1.15 to 1.54 for the LV, and it ranged from 1.34 to 1.84 for the RV. The phase‐averaged volume of the LV ranged from 191 to 440 cc, and the phase‐averaged volume of the RV ranged from 92 to 287 cc. Figure 5 plots the candlestick charts of the LV and RV volumes for each patient, which shows the minimum, maximum, 10% and 90% phase values in a cardiac cycle.

**Table 3:** Phase averaged volumes of the LV and RV for each patient as well the maximum to minimum ratio in one cardiac cycle. (LV: left ventricle; RV: right ventricle).

Patient #	LV		RV	
	Average volume (cc)	Max/min ratio	Average volume (cc)	Max/min ratio
1	243.30	1.35	199.54	1.51
2	439.51	1.15	286.88	1.34
3	243.92	1.22	232.08	1.36
4	250.05	1.37	178.20	1.60
5	258.28	1.38	116.87	1.69
6	265.52	1.54	161.41	1.64
7	374.03	1.26	110.37	1.68
8	253.06	1.30	91.76	1.46
9	241.23	1.39	127.49	1.70
10	191.25	1.41	122.48	1.84

**Fig. 6:**
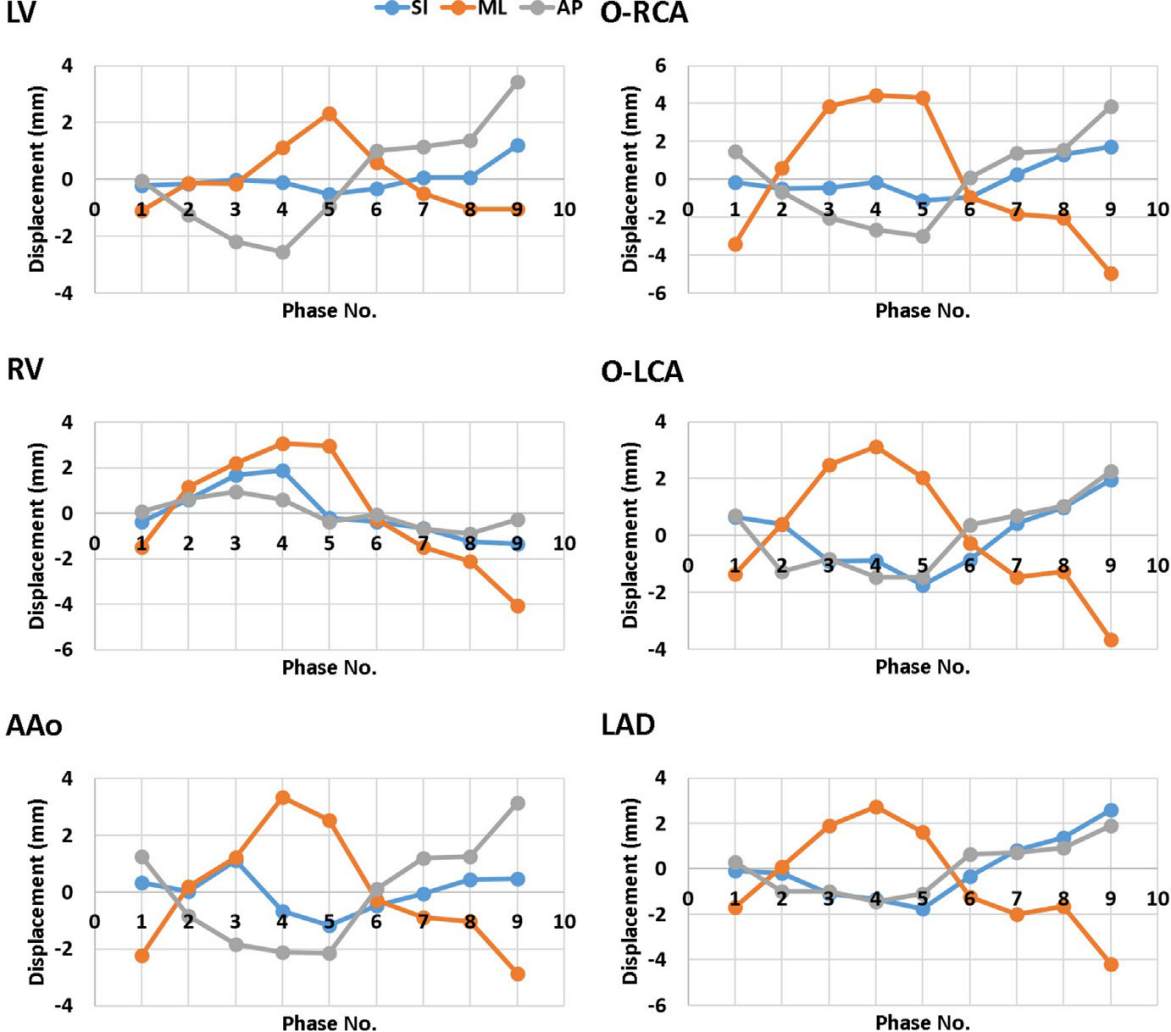
ROI centroid displacement from the average position in each phase for an example patient. (AAo: ascending aorta; LV: left ventricle; RV: right ventricle; O‐LCA: ostium of left coronary artery; O‐RCA: ostium of right coronary artery; LAD: left anterior descending artery).

**Fig. 7:**
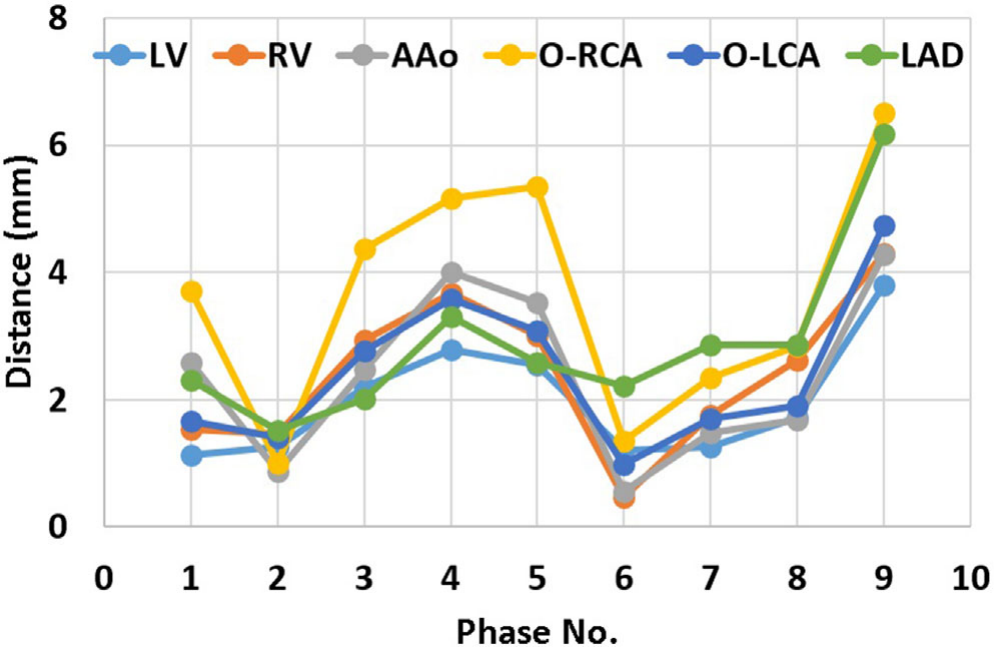
ROI centroid distance from the average position in each phase for an example patient. (AAo: ascending aorta; LV: left ventricle; RV: right ventricle; O‐LCA: ostium of left coronary artery; O‐RCA: ostium of right coronary artery; LAD: left anterior descending artery).

One patient is used as an example to demonstrate the motion in each phase. As shown in Fig. 6, this patient had motions within 6 mm from the average position in every direction. The relative motions between each structure are not trivial. The distances from the average position in each phase for the patient were within 7 mm as depicted in Fig. 7.

## 4 DISCUSSION

With a set of ten patients undergoing TAVR who received cardiac gated CT under voluntary breath‐hold, our study showed that the studied substructures had displacements with a magnitude ranging from 0 to 7.13 mm in all directions. The majority (91%) of the displacements were within 2.5 mm relative to the average positions. The composite distances ranged from 0.20 to 7.85 mm, with 91% of the distances <4 mm and 97% of the distances <5 mm. With the assumption that the breath‐hold was perfect during the imaging process, the studied motion was the cardiac motion decoupled from the respiratory motion.

As previous studies found that cardiac motion was asymmetrical,^11^, ^12^, ^13^ it is, therefore, important to study the motion of substructures of the heart. Using CT images with retrospective electrocardiographic (ECG) gating of seventeen patients, Tan et al. quantified the displacement of cardiac substructures including the anterior myocardial territory (AMT), LV, and coronary arteries during a normal cardiac cycle.^12^ They found the average displacement of the AMT and LV was 1.2–2.7 mm in the anterior and right directions, 4.3–7.8 mm in left and posterior directions, and 4.9–6.3 mm in superior and inferior directions. For the coronary arteries, the average displacement was 2.8–5.9 mm in the anterior–posterior direction, 3.5–6.6 mm in left–right direction, and 3.8–5.3 mm in the superior–inferior direction. In summary, an average of 3–8 mm motion in all directions was observed for the studied structures. In our study, the reported motions were based on the phase position relative to the average position, instead of comparing the end‐diastolic to the end‐systolic phases. When motion management tools (4‐dimensional, gating, compression, breath‐hold, etc.) are used, radiation therapy treatments are typically planned on reference CT images, which could be average CT or free‐motion CT. Therefore, we think our approach of comparing phase positions to average positions is more relevant to treatment planning of radiotherapy.

For structures like the LV and RV, the centroid displacement describes very little of their motions. In contrast, the contraction and expansion dominates their motion. Therefore, in our study, the volume changes were analyzed to supplement the centroid displacement. The coronary arteries were defined by landmark points, which were more precise in describing their motions.

The current workflow^21^ for treating VT with SBRT uses a respiratory phase‐correlated 4‐dimensional CT (4D‐CT) for contouring the internal target volume (ITV). More than one cardiac cycles exist within one respiratory cycle. The average positions of cardiac substructures are captured in such simulation CTs. While the prescribed dose is high (25 Gy in one fraction), it is still feasible to implement breath‐control methods or breath‐gating method as shown in single fraction lung SBRT treatments.^22^ In this case, only motion of cardiac substructures should be taken into consideration, potentially reducing targeted volume.

There is an increasing interest in doses to heart substructures during breast and lung radiotherapy. The current clinical standard is to use the mean heart dose as a planning constraint. However, it is shown that doses to the substructures such as left ventricle and coronary arteries are better predictors to adverse cardiac events, and mean heart doses do not correlate the substructure doses well.^14^, ^15^, ^16^, ^17^ It is, therefore, important to limit doses to heart substructures when they are spared as OARs. There is increase in dosimetric uncertainty due to the nonhomogeneous cardiac motion coupled with the respiratory motion. Our study helps understanding the cardiac motion decoupled from the respiratory motion, which may further lead to better treatment planning strategies for heart protection in breast and lung radiotherapy.

This study is conducted with an existing registry with patients undergoing TAVR, which is not a good representative of the general public or patients with VT, lung cancer, or breast cancer. In a study with 20 patients randomly selected from those who underwent CT‐based angiography, Wang et al.^23^ found the mean displacements of the heart were 6.4 and 2.5 mm in the SI and ML directions, and the mean displacements of the left anterior descending artery (LAD) were 2.6 and 2.3 mm in the ML and AP direction during deep inspiration breath‐hold. The patient diagnoses were not stated in their study. With ten patients receiving loco‐regional radiotherapy for breast cancer, Jagsi et al. studied the positional reproducibility of LAD under active breath control.^24^ They reported that the long term reproducibility of the LAD position was 3, 6, and 4 mm in the AP, SI, and ML directions at end expiration, and long term reproducibility was 3, 7, and 7 mm in the AP, SI, and ML directions at deep inspiration breath‐hold. In their study, the patients were scanned with a radiotherapy simulation CT, and the position reproducibility were studied using an average‐to‐average comparison instead of true 4D methods. Similarly, Qi et al. reported respiration induced heart motion with 20 breast cancer patients using respiratory phase‐correlated 4D‐CT and concluded that the LAD motion was up to 9 mm during free breathing.^25^

There is limited knowledge on how to incorporate true 4D heart motion into radiotherapy treatment planning. This is becoming increasingly important as radiotherapy, especially SBRT, is now being used to treat substructures of the heart. The high prescription doses and location precision demand better understanding of cardiac motion — with or without respiratory motion management. As a preliminary study, our results warrant further investigation in full motion and dosimetric modeling.

## 5 CONCLUSION

In conclusion, cardiac motion is variable but mostly within 5 mm. When a cardiac substructure is the radiation treatment target, an ITV should be drawn using the best available 4D imaging modality. If advanced imaging equipment is not available or if cardiac substructures are considered as organs at risk during radiotherapy, it is recommended to have a 5 mm expansion to account for cardiac motion. Future study includes full assessment on heart motion and its dosimetric impact.

## CONFLICT OF INTEREST

There is no conflict interest.
